# Alcohol use, intimate partner violence, and HIV sexual risk behavior among young people in fishing communities of Lake Victoria, Uganda

**DOI:** 10.1186/s12889-021-10595-1

**Published:** 2021-03-19

**Authors:** Stephen Ojiambo Wandera, Nazarius Mbona Tumwesigye, Eddy J. Walakira, Peter Kisaakye, Jennifer Wagman

**Affiliations:** 1 Department of Population Studies (DPS), School of Statistics and Planning (SSP), College of Business and Management Sciences (CoBAMS), Makerere University, Kampala, Uganda; 2 Postdoctoral Research Fellow, Fogarty International Center of the National Institutes of Health (NIH) and the University of California Global Health Institute (UCGHI), San Francisco, USA; 3 Department of Epidemiology and Biostatistics, School of Public Health, College of Health Sciences, Makerere University, Kampala, Uganda; 4 Department of Social Work and Social Administration, School of Social Sciences, College of Humanities and Social Sciences, Makerere University, Kampala, Uganda; 5 School of Public Health, University of California, Los Angeles (UCLA), Los Angeles, California USA

**Keywords:** Alcohol abuse, AUDIT, Intimate partner violence, Physical violence, Emotional violence, Sexual violence, Human Immuno-deficiency virus

## Abstract

**Background:**

Few studies have investigated the association between alcohol use, intimate partner violence, and HIV sexual risk behavior among young people in fishing communities from eastern and central Uganda. Therefore, we aimed to determine the association between alcohol use, intimate partner violence, and HIV sexual risk behavior among young people in the fishing communities on the shores of Lake Victoria, in Uganda.

**Methods:**

We conducted multivariable logistic regression analyses of HIV risk behavior using cross-sectional data from 501 young people from Mukono (Katosi landing site) and Namayingo districts (Lugala landing site).

**Results:**

Almost all (97%) respondents reported at least one HIV risk behavior; more than half (54%) reported engagement in three or more HIV risk behaviors. Results from the adjusted model indicate that alcohol use, working for cash or kind, being married, and having multiple sexual partners increased the odds of HIV risk behavior. IPV was not associated with HIV risk behavior.

**Conclusion:**

Interventions to promote consistent condom use and fewer sexual partnerships are critical for young people in the fishing communities in Uganda.

**Supplementary Information:**

The online version contains supplementary material available at 10.1186/s12889-021-10595-1.

## Background

HIV remains a public health concern [1–3]. Despite, tremendous efforts made in reducing the epidemic, 1.7 million people were living with HIV (PLHIV) in 2019 [4]. Sub-Saharan Africa (SSA) is the region with the highest global HIV prevalence. Southern and Eastern SSA are the regions most burdened by the epidemic and have the largest number of people living with HIV in the world [5]. UNAIDS estimates there were 20.7 million PLHIV in 2019 in the Eastern and Southern regions of SSA. The 2016–2017 Uganda Population-Based HIV Impact Assessment (UPHIA) estimated about 1.2 million adults to be living with HIV in the country [6].

HIV infection was first described in Uganda in 1985 in a fishing community [7]. Since then, studies have consistently documented that fishing populations are disproportionately burdened by HIV, relative to the general population in the country [8–12]. Specifically, HIV prevalence in fishing communities is estimated to be 5–7 times higher than in the general population [13]. One study estimated the HIV prevalence among people aged 18–24 years in fishing communities along the shores of Lake Victoria to be 12% in men and 26% in women [10]. Despite the high burden of HIV infection in Uganda’s fishing communities, HIV prevention and treatment strategies have not effectively reached all high-risk individuals in these communities [10, 14]. One of the largest barriers to HIV service access in Uganda’s fishing communities is that these areas are characterized by complex sexual networks that include fishermen, food vendors, fish traders, alcohol brewers, and other service providers [9]. The members of this network are hard to track and follow due to their inconsistent patterns of mobility, the time they spend away from home, their pursuit of a daily cash inflow, and their risky work environments.

Another defining feature of Uganda’s fishing communities is the high level of availability and use of alcohol, a well-established risk factor for HIV globally [15, 16] and among young people in fishing communities [17]. Alcohol use impairs judgment, reduces one’s ability to negotiate for safer sex, and lowers inhibitions [10]. Kuteesa and colleagues (2020) observe several drivers of alcohol use among fishing populations including work-related stress, mobility, geographical remoteness, and limited regulation of alcohol sales [17]. Evidence from fishing communities in Koome Islands in Mukono District in Uganda indicates that lower education, smoking, and depression are all associated with alcohol misuse [17]. In fishing communities in Tanzania, alcohol consumption is associated with higher odds of contracting HIV [12]. Relatedly, people employed in alcohol-related businesses (e.g., working in bars) in fishing communities around Lake Victoria are at greater risk of HIV infection [18].

Intimate partner violence (IPV), including physical and sexual abuse, is a risk factor for HIV acquisition [14, 19], including in fishing communities in Rakai, Uganda [20]. Sexually or physically abused women are less likely to be able to negotiate safer sex with a partner and are more likely to engage in sexual relations in exchange for money or gifts [21]. In the event of a divorce, Kher (2008) notes that women may become vulnerable and susceptible to violence since they lack financial independence and may need to stay in unsafe relationships for survival [14].

Several demographic, socio-economic, and behavioral factors are associated with sexual HIV risk among Uganda’s fishing communities. Asiki et al. (2011) observe that age, occupation, relationship to head of household, knowledge of partner’s HIV status, alcohol consumption, inconsistent condom use, marital status, being away from home for more than two nights in a month, gift exchange for sex, multiple sexual partners and a recent history of STIs were associated with sexual HIV risk among fishing communities of Lake Victoria in Uganda [10].

Age is a key variable that is associated with sexual HIV risk behavior [13, 22, 23]. Kiwanuka et al. (2014) report that HIV incidence is higher among younger people (age 18–24 years) than those age 30 years and older, in fishing communities around Lake Victoria in Uganda [23]. Adolescents are more susceptible to drug and substance abuse, sexual HIV risk behaviors [17]. Studies that focus on fisherfolk are timely because fishing populations are more vulnerable than other risky sub-groups. Kissling and colleagues (2005) observe that sexual HIV risk infection was higher among fisherfolk than other high-risk populations in DRC, Kenya, and Uganda, including sex workers, prisoners, migrant workers, men who have sex with men, military, long-distance truck drivers and drug users. In addition, alcohol consumption is common among young people. They often use alcohol to cope with daily stresses in life makes hence becoming more vulnerable through increased sexual risk taking behaviors [10, 15].

Multiple sexual partnerships among men is a sexual HIV risk behavior factor. This is higher in fishing communities than in agrarian or trading communities in Rakai Community Cohort Study (RCCS) [24]. This finding resonates with Kapesa et al. (2018), who found a similar result – higher odds of being HIV infected among fishing communities around Lake Victoria, northwestern Tanzania (three times higher) than among farming communities. Sexual HIV risk was also observed to be associated with lower levels of education, inconsistent use of the condom, and being older [12]. Results from eight fishing communities in Uganda also indicate higher sexual HIV risk among lower educated people, women, divorced or in multiple sexual relations, and users of alcohol or illicit drugs before sexual encounters [22, 23].

Some studies have included fishing communities in Gerenge [25], Kasenyi & Kigungu [18], and Rakai district [20, 26–28], in central Uganda and Kasensero from southwestern Uganda [29]. Despite the well-established burden of HIV in this setting, there is limited research exploring the correlates of sexual HIV risk factors in fishing communities among adolescents and young people in Mukono and Namayingo Districts of Uganda [9, 10, 30]. This paper fills this gap by examining sexual HIV risk factors among young people in the age group 15–24 years in fishing populations in Mukono and Namayingo districts, Uganda. Specifically, we investigate whether alcohol use and IPV influence sexual HIV risk. We focus on this specific age group because they are at the greatest risk of incident HIV [8, 18]. Our contribution to the field of sexual HIV risk is that this paper utilizes data from fishing communities to shed light on the correlates of sexual HIV risk among young people (15–24 years) in Uganda.

## Data & Methods

### Study design

A cross-sectional survey was conducted in two fishing communities in Eastern Uganda. Initially, a sequential explanatory mixed methods research design was planned; a quantitative survey to be followed by a qualitative exploration of quantitative findings. Due to the COVID-19 lockdown and travel restrictions that were enacted in Uganda on March 18, 2020, data collection stalled until it could safely be resumed in July 2020. This disruption resulted in a need to adapt the study design, leading us to concurrently collect our qualitative and quantitative data. This manuscript presents findings from the quantitative survey component.

### Sampling procedures

We selected two fishing communities along the shores of Lake Victoria for inclusion in our study: Lugala from Namayingo District (Eastern Uganda), and Katosi landing site from Mukono District (Central Uganda). The rationale for selecting these areas was the scarcity of research evidence from these sites. We used the 2014 Uganda Population and Housing Census (UPHC) sampling frame by the Uganda Bureau of Statistics (UBOS) to select sub-counties, parishes, and enumeration areas, which were fishing communities. For Mukono District, we randomly selected Katosi Town Council (formerly belonged to Ntenjeru Sub-county, until 2019), Nsanja Parish, and eight enumeration areas. For Namayingo District, we selected Banda Sub-county, Lugala Parish, and eight enumeration areas. At the time of data collection, one of the villages was submerged from Lake Victoria flooding, so it was excluded. In total there were seven enumeration areas. At each enumeration area, a sampling frame of eligible households was constructed with the aid of local council leaders from where study participants were selected.

Using Kish’s formula as cited elsewhere [31], we estimated a sample size of 202 participants per study site and a total sample size of 404 young people (15–24 years) from the two study sites. But in actual practice, the total sample size was 501. We aimed to sample nearly the same number of participants from each sampled district because we did not have the population sizes of the landing sites since these are highly migrant and mobile populations [32]. For the sample size calculation, we made an assumption of 50% HIV risk behavior obtained from a recent study [20], implying that *p* = 0.50 and *q* = 0.50, confidence level of 95%, error term of 10%, design effect of 2, and non-response of 5%. The final selection of study participants was done using simple random sampling. A pre-test of data collection tools was conducted at the Ggaba fish landing site on the shores of Lake Victoria in Kampala district in June 2020. We were able to revise the flow of few survey questions and identify those that were not clear to the respondents in terms of translation. Most of the survey questions were fine. Therefore, data collection was conducted in July 2020.

### Inclusion and exclusion criteria

Men and women age 15–24 years with a history of sexual activity were eligible for participation. Participants without a history of sexual activity were excluded because most key questions focused on IPV and HIV risk behavior and were exclusive for those who have never had a sexual partner in their lifetime. We used screening questions on the consent form that indicated whether the potential respondents had ever had sex in their lifetime.

### Data collection and management

Quantitative survey data were collected by research assistants using electronic tablets using an Open Data Kit (ODK) or Survey CTO platform [33]. A survey questionnaire was used (Supplementary File 1). Research assistants were trained for three days (July 6–8, 2020). Trained research assistants collected the survey data using the personal interview method. The selected research assistants knew the local languages (Lusamia, Lusoga, Luganda). Data collection was conducted in the local languages using translated survey tools. We performed a back-and-forth translation from English to the local languages. First, the tools were translated from English to the local dialects by professional native language speakers. Second, during the training of research assistants and role-plays, the translated tools were used. During the pre-test, language issues were checked. During the de-briefing with research assistants, translations were revised. When the research assistants arrived at the households, they requested a secluded place to ensure privacy. All data were exported to STATA for statistical analysis [34].

### Variables and measures

#### Outcome variable

HIV sexual risk behavior was measured using the HIV-Risk Screening Instrument (HRSI) [35]. The HSRI contains 10- item binary questions about multiple sexual partnerships, condom use, sexually transmitted infections (STIs), transactional sex, and substance use. Although the instrument was designed for healthcare settings, it was recommended for testing and application to the general population.

During exploratory data analyses, HIV risk measures were tested for correlation. Two pairs of the ten items were strongly correlated. First, items four and ten (respondent’s self-reported STIs and partner’s self-reported STIs respectively) had a positive correlation (r = 0.49). Second, items seven and eight (injecting drug use by respondent and partner’s injecting drug use respectively) were also positively correlated (r = 0.44). The reliability test score was 0.51.

An aggregate score of HIV risk was generated from the 10-items (mean HIV risk score = 2.87, standard deviation (SD) = 1.61, range = 0–10), for use in additional statistical analyses. Exploratory data analysis using a histogram portrayed a normal distribution of the HIV risk score. The minimum score was 0 and the maximum was 8. The HRSI recommended that a threshold of participating in at least one HIV risk item is sufficient for high-risk populations [35]. However, 97% reported at least one HIV risk behavior. This would create a common outcome. Therefore, we created a binary categorical variable for HIV risk behavior from the HIV risk score at the mean HIV risk score of 3. HIV risk behavior was recorded as participating or reporting three or more of the ten HIV risk screening items or behaviors.

#### Explanatory variables

IPV was measured using validated screening tools: the Hurt, Insult, Threaten, and Scream (HITS), the Woman Abuse Screening Tool (WAST), and the Abuse Assessment Screen (AAS) tools [36–38]. For men, the WAST questions were modified to reflect the perpetuation of violence. The HITS tool has four questions with the following response categories (1 = Never, 2 = Rarely, 3 = Sometimes, 4 = Fairly often, 5 = Frequently):

- How often did/does your partner physically **hurt** you?
- How often did/does your partner **insult** you or talk down to you?
- How often did/does your partner **threaten** you with harm?
- How often did/does your partner **scream** or curse you?

The four items were reliable measures for IPV (Chronbach’s alpha = 0.88). The mean HITS score was 7.2 (SD 4.1, range = 4–20). There was a strong positive correlation among the four items. A score of 10.5 and higher is regarded as a positive response to IPV [38, 39]. Therefore, we created a binary variable for HITS to be a yes, if the HITS score was 10.5 and higher and to be a no, otherwise.

The WAST tool has eight questions, which are also described elsewhere [36, 38, 40].

- In general, how would you describe your relationship with your partner?
- Do you and your partner work out arguments with great difficulty, some difficulty, or no difficulty?
- Do arguments ever result in you feeling down or bad about yourself?
- Do arguments ever result in hitting, kicking, or pushing?
- Do you ever feel afraid/frightened by what your partner says or does?
- Has your partner ever abused you physically?
- Has your partner ever abused you emotionally?
- Has your partner ever abused you sexually?

The first two questions (a-b) are regarded as the WAST- short form (WAST-SF). The first question (a) has the following response categories (1 = No tension, 2 = Some tension, 3 = A lot of tension). The second question (b) uses the response categories (1 = No difficulty, 2 = Some difficulty, 3 = A lot of difficulty). The last six questions (c-h) have the following response categories (1 = Never, 2 = Sometimes, 3 = Often). Exploratory analyses showed that the eight items were reliable (Cronbach’s alpha 0.89). The mean WAST score was 11.56 (SD 4.54, minimum 8, and maximum 24). To generate an IPV variable from the eight items, it is recommended that a score of 13 and higher (range = 8–24) is used to denote IPV or abuse [36, 38, 40]. Therefore, we generated a binary WAST variable to measure IPV (yes = WAST score > 13 and no = WAST score 8–12).

For the AAS tool, we used two binary questions instead of five questions [38]. The two questions were:

- In the past 12 months, were you emotionally or physically abused by your partner?
- In the last 12 months, has anyone or your partner forced you to have sexual activities against your will?

We excluded three questions that were already captured in the HITS and WAST tools. For the AAS tool, any positive response to any question denotes IPV [38]. Therefore, we created a binary IPV measure from the two questions if a respondent responded in the affirmative (yes) to any of the two binary questions.

Alcohol use was measured using the WHO’s Alcohol Use Disorder Identification Tool (AUDIT) [41–50]. It has 10 questions (response categories from 0 to 4). An AUDIT score was generated by a summation of all ten items. The minimum score was 0 and the maximum was 40. The mean AUDIT score was 2.5 (SD = 5.5) because it was affected by the non-drinkers. Studies in Australia recommend that to create AUDIT categories, AUDIT scores of 0 is for non-drinkers, 1–7 denotes “low-risk drinkers”, 8–12 denotes “at-risk drinkers”, 13–19 represents “high-risk drinkers, and 20-40 means “dependent drinkers” [44]. Therefore, we created a categorical AUDIT variable following these guidelines.

Demographic and socio-economic variables included district of residence (Mukono and Namayingo), sex (male or female), age group [15–24], an education level (None, Primary, Secondary or Higher), employment status (Yes, No), religious affiliation (Catholic, Anglican, Pentecostal, Muslim and others), children ever born or ever given birth or fathered a child (yes or no), and marital status (never married, married, living together, formerly married). Partners’ characteristics included education (None, Primary, Secondary or Higher), how often the partner got drunk with alcohol (yes or no), and whether their partners accused them of having other sexual partners in their lifetime or recently (yes or no).

### Statistical analysis

Frequency distributions were used to analyze the descriptive characteristics of the respondents. Chi-square tests were used to measure the association between HIV risk and alcohol use, measures of IPV, and selected explanatory variables. Multivariate logistic regression was used to estimate the relationship between HIV sexual risk behavior as a binary outcome and alcohol use, IPV, and variables that had a 10% or less significant association with the outcome variable [34, 51].

Two models were estimated. First, HIV risk was regressed against alcohol use and IPV as primary covariates (unadjusted model). Second, HIV sexual risk behavior was regressed against the two primary covariates (alcohol use and IPV) while controlling for demographic (age, sex, marital status, children ever born), socio-economic factors (education level, employment status), and behavioral factors. Regression diagnostics included the use of the link test to determine the goodness of fit of the models. Also, pairwise correlation and collin commands were conducted to ascertain possibilities of multicollinearity among the covariates.

## Results

### Descriptive characteristics

Table 1 shows the descriptive characteristics of the respondents. There was an even distribution of respondents from Mukono and Namayingo districts. More than half (61%) were female, and three-quarters (75%) were age 20–24 years (mean age of 21.1 years, a standard deviation of 2.4 years).

**Table 1:** Descriptive characteristics of the respondents, alcohol use, IPV and HIV sexual risk behavior

Variables	Frequency (n)	Percent (%)
**District**		
Mukono	251	50.1
Namayingo	250	49.9
**Sex**		
Female	304	60.7
Male	197	39.3
**Age category**	**(mean = 21.1, SD = 2.4)**	
15–19	126	25.1
20–24	375	74.9
**Education level**		
None	79	15.8
Primary	227	45.3
Secondary or higher	195	38.9
**In the last 12 months, have you done any work for which you received a payment, in cash or in kind**		
No	178	35.5
Yes	323	64.5
**What is your main source of livelihood?**		
Fishing	86	17.2
Student	23	4.6
Farming	88	17.6
Petty Trade	126	25.1
Others	178	35.5
**Religion**		
Catholic	198	39.5
Anglican	132	26.3
Pentecostal	80	16.0
Muslim or others	91	18.2
**Marital status**		
Never Married	94	18.8
Married	82	16.4
Living together	267	53.3
Formerly married	58	11.6
**Have you ever given birth to or fathered a child?**		
No	143	28.5
Yes	358	71.5
**In the last 12 months, have you had a sexual partner?**		
No	32	6.4
Yes	469	93.6
**Total**	**501**	**100**
**Partner’s education level**		
None	99	21.1
Primary	181	38.6
Secondary or higher	189	40.3
**How long have you been in a relationship with your current partner in complete years?**	**(mean = 1.9, SD = 0.7)**	
Less than a year	117	24.9
One to three years	250	53.3
Four years or more	102	21.7
**Partner drinks alcohol**		
No	345	73.6
Yes	124	26.4
**Accused or has other sexual partners**		
No	282	56.3
Yes	219	43.7
**Prevented HIV using two or more methods in the last 3 months**		
No	181	36.1
Yes	320	63.9
**Has comprehensive knowledge of HIV transmission and prevention**		
No	1	0.2
Yes	500	99.8
Variables	Number (n)	Percent (%)
**AUDIT score categories**	**(Mean = 2.5, SD = 5.6)**	
Non-drinkers	308	61.5
Low risk drinkers	135	26.9
At risk drinkers	28	5.6
High risk drinkers	20	4.0
Dependent drinkers	10	2.0
**Drinks alcohol**		
Non-drinkers	308	61.5
Alcohol drinkers	193	38.5
**Reported lifetime IPV by the HITS scale**	**(Mean = 7.2, SD = 4.1)**	
No	399	79.6
Yes	102	20.4
**Reported lifetime IPV by the WAST scale**	**Mean = 11.6, SD = 4.5**	
No	300	59.9
Yes	201	40.1
**Reported IPV by the AAS scale**		
No	186	37.1
Yes	315	62.9
**Reported three or more HIV sexual risk behaviors**		
No	230	45.9
Yes	271	54.1
**Total**	**501**	**100**

The majority (45%) of the young people had primary education and six in ten (65%) worked for payment in the last 12 months. A quarter (25%) of them were engaged in petty trade and a small proportion (18%) participated in fishing activities. Catholicism was the most widely practiced religion (40%). More than half (70%) were married and 12% were formerly married. Over two-thirds (72%) had ever given birth or fathered a child. Nearly all respondents (94%) had sexual partners in the last 12 months.

Forty percent of the young people had partners who had achieved secondary or higher education. More than half (53%) had lived in a relationship for between one and three years (mean duration was 1.9 years and standard deviation of 0.7 years). A quarter (26%) reported that their partners consumed alcohol. Four in ten (44%) of the respondents had or were accused of having other sexual partners by the current partner. More than half (64%) used two or more methods to prevent HIV in the last three months. Nearly all respondents (99%) had comprehensive knowledge about HIV transmission and prevention mechanisms.

Table 1 also shows the prevalence of alcohol use, IPV, and HIV risk behaviors. The mean AUDIT score was 2.5 (standard deviation of 5.6). Nearly four in ten (39%) were alcohol drinkers. Of those who drunk alcohol, a quarter (27%) were low-risk drinkers and 12% were risky drinkers.

The prevalence of IPV in the last 12 months was 21% using the HITS scale, 40% using the WAST scale, and 63% using the AAS scale. Almost all (97%) respondents reported at least one HIV risk behavior, and more than half (54%) of the respondents reported three or more HIV risk behaviors.

**Fig. 1:**
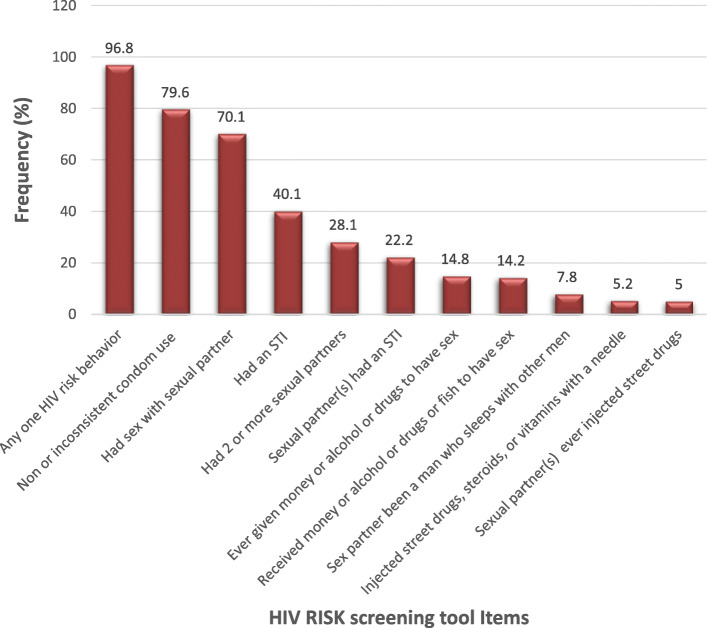
Prevalence of HIV Risk Screening Behaviors from the HRSI

Figure 1 shows the HIV risk screening tool items. Almost all (97%) of the respondents reported at least one or more HIV risk behaviors. The most common HIV risk behaviors were non or inconsistent condom use (80%), being sexually active (70%), self-reported STIs (40%), multiple sexual partnerships (28%), and self-reported STIs by partner (22%).

### Association between HIV risk and explanatory variables

Table 2 shows the association between HIV risk behaviors and selected explanatory variables. In bivariate analysis, there were no significant differences in the prevalence of three or more HIV risk behaviors by district, age category, education level, religion, and partner’s education level. The prevalence of HIV risk was higher among males compared to females (60% vs 50%; *p* = 0.04). HIV risk behavior was highest (60%; *p* = < 0.001) among respondents who worked and received payment in cash or kind and depended on fishing (67%; *p* = < 0.001) as their source of livelihood. Furthermore, HIV risk behavior was highest among respondents who were married (68%; *p* = < 0.01) and ever gave birth or fathered a child (58%; *p* = < 0.01). Those who drank alcohol reported three or more HIV risk behaviors (65%; *p* = 0.02). Respondents who prevented HIV in two or more ways had lower HIV risk behaviors.

**Table 2:** Association between HIV sexual risk behaviors and selected variables

Variables	Reported three or more HIV risk behaviors		
	**Percent (%)**	**Total (n)**	**Chi2**
**District**			0.26
Mukono	56.6	251	
Namayingo	51.6	250	
**Sex**			**0.04**
Female	50.3	304	
Male	59.9	197	
**Age category**			0.39
15–19	50.8	126	
20–24	55.2	375	
**Education level**			0.08
None	63.3	79	
Primary	55.5	227	
Secondary or higher	48.7	195	
**In the last 12 months, respondent worked for payment in cash or kind**			**< 0.001**
No	42.7	178	
Yes	60.4	323	
**What is your main source of livelihood?**			**< 0.001**
Fishing	67.4	86	
Student	26.1	23	
Farming	58.0	88	
Petty Trade	54.8	126	
Others	48.9	178	
**Religion**			0.09
Catholic	56.6	198	
Anglican	53	132	
Pentecostal	42.5	80	
Muslim or others	60.4	91	
**Marital status**			**< 0.01**
Never Married	39.4	94	
Married	68.3	82	
Living together	53.9	267	
Formerly married	58.6	58	
**Have you ever given birth to or fathered a child?**			**< 0.01**
No	44.1	143	
Yes	58.1	358	
**In the last 12 months, have you had a sexual partner?**			**0.02**
No	34.4	32	
Yes	55.4	469	
**Total**	**54.1**	**501**	
**Partner’s education level**			0.17
None	63.6	99	
Primary	54.1	181	
Secondary or higher	52.4	189	
**How long have you been in a relationship with your current partner in complete years**			0.11
Less than a year	50.4	117	
One to three years	60	250	
Four or more years	50	102	
**Partner drinks alcohol**			**0.02**
No	52.2	345	
Yes	64.5	124	
**Accused by a partner to have or has other sexual partners**			**< 0.001**
No	44.7	282	
Yes	66.2	219	
**Prevented HIV using two or more methods in the last 3 months**			**0.02**
No	60.8	181	
Yes	50.3	320	
**AUDIT score categories**			**0.002**
Non-drinkers	48.4	308	
Low risk drinkers	60	135	
At risk drinkers	60.7	28	
High risk drinkers	70	20	
Dependent drinkers	100	10	
**Drinks alcohol**			**0.001**
Non-drinkers	48.4	308	
Alcohol drinkers	63.2	193	
**Reported lifetime IPV by the HITS scale**			**0.049**
No	51.9	399	
Yes	62.7	102	
**Reported lifetime IPV by the WAST scale**			**0.015**
No	49.7	300	
Yes	60.7	201	
**Reported IPV by the AAS scale**			**0.019**
No	47.3	186	
Yes	58.1	315	
**Total**	**54.1**	**501**	

Table 2 also shows the association between HIV risk and alcohol use and IPV measures. The prevalence of HIV risk behaviors was higher among those who drunk alcohol compared to those who did not (63% vs 48%; *p* < 0.01). There was some difference in the prevalence of HIV risk among those that experienced IPV by HITS scale compared to those who did not (63% vs 52%; *p* = 0.05). The prevalence of HIV risk was higher among those who experienced IPV by the WAST scale compared to those who did not (61% vs 50%; *p* = 0.02). The prevalence of HIV risk was higher among those who experienced IPV by the AAS scale compared to those who did not (58% vs 47%; *p* = 0.02). Finally, experiencing IPV by HITS or WAST or AAS was associated with a higher prevalence of HIV risk (57% vs 47%; *p* = 0.02).

### Multivariate results

Table 3 shows the multivariable logistic regression models fit to identify factors associated with HIV sexual risk behavior. The key correlates of HIV risk were alcohol use, working for payment in cash or kind, being married, and having other sexual partners. However, IPV was associated with HIV sexual risk behavior at the bivariate level, not at the multivariate level.

**Table 3:** Multivariable regression of HIV risk on alcohol use, IPV and selected explanatory variables

	Model (1)		Model (2)	
	Unadjusted Odds Ratios (OR)	95% Confidence Intervals (CIs)	Adjusted Odds Ratios (aOR)	95% Confidence Intervals (CIs)
Drinks alcohol (rc = no)	1.83^**^	1.27–2.65	1.83^**^	1.18–2.85
Reported lifetime abuse – HITS scale+	1.56^*^	0.99–2.44		
Reported lifetime abuse – WAST scale	1.57^*^	1.09–2.25	1.09	0.69–1.72
Reported lifetime abuse – AAS scale++	1.54^*^	1.07–2.22		
What is your age in complete years?			0.99	0.90–1.09
Sex (rc = females)			1.00	0.59–1.68
**Education level of respondent (rc = None)**				
Primary			0.65	0.36–1.18
Secondary or higher			0.62	0.32–1.21
In the last 12 months, have you done any work for which you received a payment in cash or kind			2.13^**^	1.30–3.49
**Religion (rc = Catholic)**				
Anglican			0.81	0.49–1.33
Pentecostal			0.61	0.34–1.09
Muslim			1.51	0.76–3.00
Others			1.23	0.53–2.86
**Marital status (rc = never married)**				
Married			2.29^*^	1.08–4.86
Living together			1.54	0.81–2.94
Formerly married			1.56	0.65–3.74
Have you ever given birth to or fathered a child?			1.32	0.77–2.27
Accused by the current partner to have or reported having other sexual partners			2.06^**^	1.33–3.19
Prevented HIV using two or more methods in the last 3 months			0.70	0.46–1.09
**Observations**	**501**		**501**	

*+ excluded because of borderline significance; Excluded because of multicollinearity*

Alcohol use was associated with increased odds of HIV risk at bivariate (OR = 1.83, 95% CI: 1.27–2.65), and multivariate analyses (aOR = 1.83, 95% CI: 1.18–2.85) respectively. When IPV measures were added in model 2, there was no change in effect sizes (both adjusted odds ratios and significance levels) for alcohol use. Slight changes in effect sizes for alcohol use were realized when we adjusted for socioeconomic factors.

Working for payment in the last 12 months increased the odds of HIV risk (aOR = 2.13, 95% CI: 1.30–3.49). Being married was associated with increased odds (aOR = 2.29, 95% CI: 1.08–4.86) of HIV risk compared to being never married. Finally, being accused by the partner or having another sexual partner increased the odds (aOR = 2.06, 95% CI: 1.33–3.19) of HIV risk.

## Discussion

This study aimed to investigate the association between alcohol use, IPV, and HIV sexual risk behaviors among young people in fishing communities in Uganda. We found that 54% of young people reported three or more HIV risk behaviors and 97% reported at least one HIV risk behavior. Other studies report a high prevalence of HIV risk behaviors in fishing communities [52–55]. Factors associated with HIV sexual risk behavior included alcohol use, employment status, being in a marital union, and multiple sexual partnerships.

Alcohol use was associated with increased odds of HIV risk behaviors in models 1 and 2. Slight changes in effect sizes for alcohol use were realized when we adjusted for alcohol use and demographic and socioeconomic factors. As earlier stated, alcohol consumption is a risk factor for HIV infection in fishing communities [17] because it impairs judgment, reduces the ability to negotiate for safe sex [10] especially condom use, and promotes multiple sexual partnerships. From our findings, non- or inconsistent condom use was the leading (70%) HIV risk behavior and more than a quarter (28%) of the respondents reported multiple sexual partnerships. Although multiple sexual partnerships were relatively low, they could be underreported due to social desirability bias. Kuteesa and colleagues (2020) noted that alcohol abuse among fishing populations results from work stresses, mobility, geographical remoteness, and limited regulation [17]. Evidence from fishing communities in Koome Islands in Mukono district in Uganda indicates that alcohol misuse among people in the age group 15–24 years was mainly associated with lower education, and depression [17]. Similar evidence has been reported among fishing communities surrounding Lake Victoria in northwestern Tanzania [12].

Contrary to our expectation, we found that IPV was not associated with HIV sexual risk behavior [14, 19]. It is true that sexually or physically abused women are less likely to negotiate for safer sex and are more likely to engage in sexual relations in exchange for money or gifts [21, 56]. From these data, we used the WAST IPV measure. HITS and AAS had borderline statistical significance and multicollinearity. To investigate this relationship further, we conducted bivariate regressions of HIV risk behavior and each of the measures of IPV separately (HITS, WAST, and AAS) and found no significant associations (Results not presented). There is a strong body of evidence that suggests that IPV is associated with HIV risky behaviors [57–59]. Apparently, alcohol consumption is a stronger predictor of HIV sexual risk than IPV. Alcohol consumption strongly impacts IPV experiences among people within and without fishing communities [28, 60, 61]. As much as IPV was not directly related to HIV sexual risk behavior, there is a possibility of an indirect pathway. This finding is similar that of a study done in India, Haiti, and Mali which did not find an association between IPV and HIV [62]. Also, some study has reported no association between HIV status and IPV rates [63]. From our findings, we suspect under-reporting and social desirability bias regarding IPV. Perhaps, there is a need to investigate this phenomenon further using qualitative inquiries. According to the WHO bulletin, IPV has an indirect effect on HIV by fueling riskier sexual behavior [64]. From our findings, being accused of having an additional sexual partner by a current partner or reporting other sexual partners was associated with HIV sexual risk behavior.

In this study, our findings indicate that working for payment in cash or in-kind in the last 12 months increased the odds of HIV risk. Some young people engage in jobs such as working in bars, and fish trading which may lead to or exacerbate risky sexual behavior even though one earns a wage [57–59].

Being married was associated with increased odds (aOR = 2.35, 95% CI: 1.15–4.83) of sexual HIV risk compared to being never married. The literature suggests that HIV risk behavior and infection are higher among married fisher-folk who are highly mobile and stay away from their spouses during work-related mobility seasons [65, 66].

Finally, being accused by a current partner or having another sexual partner increased the odds (aOR = 2.14, 95% CI: 1.41–3.23) of HIV risk behavior. The Syndemic theory is a good explanation for this phenomenon. One risky sexual behavior has the potential to exacerbate others and therefore, influence health outcomes [67].

### Implications

Future studies should examine how patterns of alcohol use differ between males and females and could examine the associations between alcohol abuse and IPV experiences of young people in the fishing communities in Uganda.

Consistent with other fishing communities in East Africa, we found an association between alcohol use and engagement in HIV sexual risk behaviors among young people in eastern and central Ugandan fishing communities. This suggests that interventions to reduce alcohol use among young people may be an important pathway for HIV risk prevention. It may not be realistic to eliminate all forms of alcohol use and abuse from the landing sites. However, it is feasible to formulate regulations and by-laws that regulate alcohol use for young people in the fishing communities in Uganda.

Also, interventions to promote consistent condom use, reduce multiple sexual partnerships, promote faithfulness to one sexual partner, and treat sexually transmitted infections, are critical for young people in the fishing communities in Uganda. Although the Government of Uganda promotes the ABC (abstinence, be faithful, consistent condom use) and male medical circumcision, there is a need to facilitate and implement programs that address these core components of HIV prevention in the fishing communities in Mukono and Namayingo districts.

### Study strengths and limitations

The strength of this study is that it uses a cross-sectional survey to provide evidence about the association between alcohol use, intimate partner violence, and HIV risk behavior among fisherfolk from eastern and central Uganda.

Findings from this study should be interpreted considering some limitations. First, the analyses are based on cross-sectional data, which makes it difficult to infer the direction of causality between alcohol, IPV, and HIV risk. Finally, there is a possibility of under-reporting of alcohol abuse, IPV, and HIV risk measures due to stigma, social desirability bias, and trauma.

## Conclusions

HIV risk behaviors among young people in fishing communities along Lake Victoria shores are common in Mukono and Namayingo districts. Factors associated with HIV risk among young people included alcohol use, working for payment in cash or kind, being married, and having other sexual partners but not with IPV.

Interventions are needed to address HIV risk behaviors among young people in fishing communities. These should focus on reducing alcohol abuse in these communities. Future research could examine associations between alcohol abuse and the IPV experiences of young people. Interventions to promote consistent condom use, reduce multiple sexual partnerships, and treat sexually transmitted infections are critical for young people in the fishing communities in Uganda.

## Supplementary Information

**Additional file 1.**

## Data Availability

The datasets generated and analyzed during the current study are not publicly available due to confidentiality reasons but are available from the corresponding author on reasonable request.

## Abbreviations

AAS: Abuse Assessment Screen
AUDIT: Alcohol Use Disorder Identification Tool
HITS: Hurt, Insult, Threaten and Scream
HIV: Human Immune-Deficiency Virus
IPV: Intimate Partner Violence
WAST: Woman Abuse Screening Tool
Who: World Health Organization

## References

[1] Zak-Place J, Stern M (2004). Health belief factors and dispositional optimism as predictors of STD and HIV preventive behavior. J Am Coll Heal.

[2] Bourgeois AC, Edmunds M, Awan A, Jonah L, Varsaneux O, Siu W (2017). HIV in Canada-surveillance report, 2016. Can Commun Dis Rep.

[3] Bourne PA, Charles CAD (2010). Sexual behavior and attitude towards HIV testing among non-HIV testers in a developing nation: a public health concern. N Am J Med Sci.

[4] UNAIDS (2020). Global HIV & AIDS statistics - 2020 fact sheet.

[5] Abu-Raddad LJ, Barnabas RV, Janes H, Weiss HA, Kublin JG, Longini IM (2013). Have the explosive HIV epidemics in sub-Saharan Africa been driven by higher community viral load?. AIDS (London, England).

[6] Ministry of Health Uganda (2019). Uganda population-based HIV impact assessment (UPHIA) 2016–2017: final report.

[7] Serwadda D, Sewankambo NK, Carswell JW, Bayley AC, Tedder RS, Weiss RA, Mugerwa RD, Lwegaba A, Kirya GB, Downing RG, Clayden SA, Dalgleish AG (1985). Slim disease: a new disease in Uganda and its ASSOCIATION with HTLV-iii infection. Lancet.

[8] Kissling E, Allison EH, Seeley JA, Russell S, Bachmann M, Musgrave SD, Heck S (2005). Fisherfolk are among groups most at risk of HIV: cross-country analysis of prevalence and numbers infected. AIDS..

[9] Allison EH, Seeley JA (2004). HIV and AIDS among fisherfolk: a threat to ‘responsible fisheries'?. Fish Fish.

[10] Asiki G, Mpendo J, Abaasa A, Agaba C, Nanvubya A, Nielsen L, Seeley J, Kaleebu P, Grosskurth H, Kamali A (2011). HIV and syphilis prevalence and associated risk factors among fishing communities of Lake Victoria, Uganda. Sexually Transmitted Infections.

[11] Kagaayi J, Chang LW, Ssempijja V, Grabowski MK, Ssekubugu R, Nakigozi G, Kigozi G, Serwadda DM, Gray RH, Nalugoda F, Sewankambo NK, Nelson L, Mills LA, Kabatesi D, Alamo S, Kennedy CE, Tobian AAR, Santelli JS, Ekström AM, Nordenstedt H, Quinn TC, Wawer MJ, Reynolds SJ (2019). Impact of combination HIV interventions on HIV incidence in hyperendemic fishing communities in Uganda: a prospective cohort study. The Lancet HIV.

[12] Kapesa A, Basinda N, Nyanza EC, Mushi MF, Jahanpour O, Ngallaba SE (2018). Prevalence of HIV infection and uptake of HIV/AIDS services among fisherfolk in landing islands of Lake Victoria, north western Tanzania. BMC Health Serv Res.

[13] Kiwanuka N, Ssetaala A, Ssekandi I, Nalutaaya A, Kitandwe PK, Ssempiira J, Bagaya BS, Balyegisawa A, Kaleebu P, Hahn J, Lindan C, Sewankambo NK (2017). Population attributable fraction of incident HIV infections associated with alcohol consumption in fishing communities around Lake Victoria. Uganda PLOS ONE.

[14] Kher A (2008). Review of social science literature on risk and vulnerability to HIV/AIDS among fishing communities in sub-Saharan Africa.

[15] Williams EC, Hahn JA, Saitz R, Bryant K, Lira MC, Samet JH (2016). Alcohol use and human immunodeficiency virus (HIV) infection: current knowledge, implications, and future directions. Alcohol Clin Exp Res.

[16] Fritz KE, Woelk GB, Bassett MT, McFarland WC, Routh JA, Tobaiwa O (2002). The association between alcohol use, sexual risk behavior, and HIV infection among men attending Beerhalls in Harare, Zimbabwe. AIDS and Behavior.

[17] Kuteesa MO, Weiss HA, Cook S, Seeley J, Ssentongo JN, Kizindo R, et al. Epidemiology of Alcohol Misuse and Illicit Drug Use Among Young People Aged 15–24 Years in Fishing Communities in Uganda. Int J Environment Res Public Health. 2020;17(7):1-21. 10.3390/ijerph17072401 PMC7178227 32244722

[18] Seeley J, Nakiyingi-Miiro J, Kamali A, Mpendo J, Asiki G, Abaasa A, de Bont J, Nielsen L, Kaleebu P, CHIVTUM Study Team (2012). High HIV incidence and socio-behavioral risk patterns in fishing communities on the shores of Lake Victoria, Uganda. Sex Transm Dis.

[19] Ngabirano TD, Saftner MA, BJ MM. Exploring Health Behaviors in Ugandan Adolescents Living in Rural Fishing Communities. J School Nurs. 2020;1059840520947142. 10.1177/1059840520947142. 10.1177/1059840520947142 32757810

[20] Sabri B, Wirtz AL, Ssekasanvu J, Nonyane BAS, Nalugoda F, Kagaayi J, Ssekubugu R, Wagman JA (2019). Intimate partner violence, HIV and sexually transmitted infections in fishing, trading and agrarian communities in Rakai, Uganda. BMC Public Health.

[21] Kaye DK (2004). Gender inequality and domestic violence: implications for human immunodeficiency virus (HIV) prevention. Afr Health Sci.

[22] Kiwanuka N, Ssetaala A, Mpendo J, Wambuzi M, Nanvubya A, Sigirenda S, Nalutaaya A, Kato P, Nielsen L, Kaleebu P, Nalusiba J, Sewankambo NK (2013). High HIV-1 prevalence, risk behaviours, and willingness to participate in HIV vaccine trials in fishing communities on Lake Victoria, Uganda. J Int AIDS Soc.

[23] Kiwanuka N, Ssetaala A, Nalutaaya A, Mpendo J, Wambuzi M, Nanvubya A, Sigirenda S, Kitandwe PK, Nielsen LE, Balyegisawa A, Kaleebu P, Nalusiba J, Sewankambo NK (2014). High incidence of HIV-1 infection in a general population of fishing communities around Lake Victoria, Uganda. PLoS One.

[24] Chang LW, Grabowski MK, Ssekubugu R, Nalugoda F, Kigozi G, Nantume B, Lessler J, Moore SM, Quinn TC, Reynolds SJ, Gray RH, Serwadda D, Wawer MJ (2016). Heterogeneity of the HIV epidemic in agrarian, trading, and fishing communities in Rakai, Uganda: an observational epidemiological study. Lancet HIV.

[25] Sileo KM, Kintu M, Chanes-Mora P, Kiene SM (2016). "such behaviors are not in my Home Village, I got them Here": a qualitative study of the influence of contextual factors on alcohol and HIV risk behaviors in a fishing community on Lake Victoria, Uganda. AIDS Behav.

[26] Mullinax M, Grilo SA, Song XS, Wagman J, Mathur S, Nalugoda F, Lutalo T, Santelli J (2017). HIV-risk behaviors of men who perpetrate intimate partner violence in Rakai, Uganda. AIDS Educ Prev.

[27] Wagman JA, King EJ, Namatovu F, Kiwanuka D, Kairania R, Semanda JB, Nalugoda F, Serwadda D, Wawer MJ, Gray R, Brahmbhatt H (2016). Combined intimate partner violence and HIV/AIDS prevention in rural Uganda: design of the SHARE intervention strategy. Health Care Women Int.

[28] Zablotska IB, Gray RH, Koenig MA, Serwadda D, Nalugoda F, Kigozi G, Sewankambo N, Lutalo T, Mangen FW, Wawer M (2009). Alcohol use, intimate partner violence, sexual coercion and HIV among women aged 15-24 in Rakai, Uganda. AIDS And Behavior.

[29] Lubega M, Nakyaanjo N, Nansubuga S, Hiire E, Kigozi G, Nakigozi G (2015). Understanding the socio-structural context of high HIV transmission in kasensero fishing community, South Western Uganda. BMC Public Health.

[30] Mafigiri R, Matovu JKB, Makumbi FE, Ndyanabo A, Nabukalu D, Sakor M, Kigozi G, Nalugoda F, Wanyenze RK (2017). HIV prevalence and uptake of HIV/AIDS services among youths (15–24 years) in fishing and neighboring communities of Kasensero, Rakai District, South Western Uganda. BMC Public Health.

[31] Kish L (1965). Sampling organizations and groups of unequal sizes. Am Sociol Rev.

[32] Breuer C, Bloom B, Miller AP, Kigozi G, Nakyanjo N, Ddaaki W, Nalugoda F, Wagman JA (2019). "the bottle is my wife": exploring reasons why men drink alcohol in Ugandan fishing communities. Social work in public health.

[33] SurveyCTO (2019). SurveyCTO: SurveyCTO.

[34] StataCorp (2015). Stata Statistical software StataCorp.

[35] Gerbert B, Bronstone A, McPhee S, Pantilat S, Allerton M (1998). Development and testing of an HIV-risk screening instrument for use in health care settings. Am J Prev Med.

[36] Arkins B, Begley C, Higgins A (2016). Measures for screening for intimate partner violence: a systematic review. J Psychiatr Ment Health Nurs.

[37] Paterno MT, Draughon JE (2016). Screening for intimate partner violence. J Midwifery Women's Health.

[38] Rabin RF, Jennings JM, Campbell JC, Bair-Merritt MH (2009). Intimate partner violence screening tools: a systematic review. Am J Preventive Med.

[39] Sherin KM, Sinacore JM, Li XQ, Zitter RE, Shakil A (1998). HITS: a short domestic violence screening tool for use in a family practice setting. Fam Med.

[40] Brown JB, Lent B, Schmidt G, Sas G (2000). Application of the woman abuse screening tool (WAST) and WAST-short in the family practice setting. J Family Pract.

[41] Bohn MJ, Babor TF, Kranzler HR (1995). The alcohol use disorders identification test (AUDIT): validation of a screening instrument for use in medical settings. J Stud Alcohol.

[42] Bowring AL, Gouillou M, Hellard M, Dietze P (2013). Comparing short versions of the AUDIT in a community-based survey of young people. BMC Public Health.

[43] Bush K, Kivlahan DR, McDonell MB, Fihn SD, Bradley KA (1998). The AUDIT alcohol consumption questions (AUDIT-C): an effective brief screening test for problem drinking. Ambulatory care quality improvement project (ACQUIP). Alcohol use disorders identification test. Arch Intern Med.

[44] Calabria B, Clifford A, Shakeshaft AP, Conigrave KM, Simpson L, Bliss D (2014). Identifying Aboriginal-specific AUDIT-C and AUDIT-3 cutoff scores for at-risk, high-risk, and likely dependent drinkers using measures of agreement with the 10-item Alcohol Use Disorders Identification Test. Addict Sci Clin Pract.

[45] Gordon AJ, Maisto SA, McNeil M, Kraemer KL, Conigliaro RL, Kelley ME, Conigliaro J (2001). Three questions can detect hazardous drinkers. J Family Pract.

[46] Higgins-Biddle JC, Babor TF (2018). A review of the alcohol use disorders identification test (AUDIT), AUDIT-C, and USAUDIT for screening in the United States: past issues and future directions. Am J Drug Alcohol Abuse.

[47] Hodgson R, Alwyn T, John B, Thom B, Smith A (2002). The FAST Alcohol Screening Test. Alcohol and alcoholism (Oxford, Oxfordshire).

[48] Pradhan B, Chappuis F, Baral D, Karki P, Rijal S, Hadengue A (2012). The alcohol use disorders identification test (AUDIT): validation of a Nepali version for the detection of alcohol use disorders and hazardous drinking in medical settings. Substance abuse treatment, prevention, and policy.

[49] Santis R, Garmendia ML, Acuna G, Alvarado ME, Arteaga O (2009). The alcohol use disorders identification test (AUDIT) as a screening instrument for adolescents. Drug Alcohol Depend.

[50] Saunders JB, Aasland OG, Babor TF, de la Fuente JR, Grant M (1993). Development of the Alcohol Use Disorders Identification Test (AUDIT): WHO Collaborative Project on Early Detection of Persons with Harmful Alcohol Consumption--II. Addiction (Abingdon, England).

[51] Vintzileos AM, Ananth CV (2010). How to write and publish an original research article. Am J Obstet Gynecol.

[52] Nkomazana N, Maharaj P (2014). Perception of risk of HIV infections and sexual behaviour of the sexually active university students in Zimbabwe. SAHARA-J: Journal of Social Aspects of HIV/AIDS.

[53] Price JT, Rosenberg NE, Vansia D, Phanga T, Bhushan NL, Maseko B (2018). Predictors of HIV, HIV Risk Perception, and HIV Worry Among Adolescent Girls and Young Women in Lilongwe, Malawi. Journal of acquired immune deficiency syndromes (1999).

[54] Sileo KM, Kintu M, Kiene SM (2018). The intersection of intimate partner violence and HIV risk among women engaging in transactional sex in Ugandan fishing villages. AIDS Care.

[55] Stringer EM, Sinkala M, Kumwenda R, Chapman V, Mwale A, Vermund SH (2004). Personal risk perception, HIV knowledge and risk avoidance behavior, and their relationships to actual HIV serostatus in an urban African obstetric population. J Acquired Immune Deficiency Syndromes (1999).

[56] Raj A, Santana MC, Marche AL, Amaro H, Cranston K, Silverman JG (2006). Perpetration of intimate partner violence associated with sexual risk behaviors among young adult men. Am J Public Health.

[57] Dunkle KL, Jewkes RK, Nduna M, Levin J, Jama N, Khuzwayo N, Koss MP, Duvvury N (2006). Perpetration of partner violence and HIV risk behaviour among young men in the rural eastern cape. South Africa AIDS.

[58] González-Guarda RM, Florom-Smith AL, Thomas T (2011). A Syndemic model of substance abuse, intimate partner violence, HIV infection, and mental health among Hispanics. Public Health Nurs.

[59] Karamagi CAS, Tumwine JK, Tylleskar T, Heggenhougen K (2006). Intimate partner violence against women in eastern Uganda: implications for HIV prevention. BMC Public Health.

[60] Morojele NK, Kachieng’a MA, Mokoko E, Nkoko MA, Parry CDH, Nkowane AM, Moshia KM, Saxena S (2006). Alcohol use and sexual behaviour among risky drinkers and bar and shebeen patrons in Gauteng province, South Africa. Soc Sci Med.

[61] Schulkind J, Mbonye M, Watts C, Seeley J (2016). The social context of gender-based violence, alcohol use and HIV risk among women involved in high-risk sexual behaviour and their intimate partners in Kampala, Uganda. Culture, Health & Sexuality.

[62] Harling G, Msisha W, Subramanian SV (2010). No association between HIV and intimate partner violence among women in 10 developing countries. PLoS One.

[63] Gielen AC, McDonnell KA, O'Campo PJ (2002). Intimate partner violence, HIV status, and sexual risk reduction. AIDS Behav.

[64] WHO (2004). Violence against women and HIV: critical intersections (intimate partner violence and HIV/AIDS). Bull World Health Organ.

[65] Kwena ZA, Camlin CS, Shisanya CA, Mwanzo I, Bukusi EA (2013). Short-term mobility and the risk of HIV infection among married couples in the fishing communities along Lake Victoria. Kenya PLoS One.

[66] Kwiringira JN, Ariho P, Zakumumpa H, Mugisha J, Rujumba J, Mugisha MM (2019). Livelihood risk, culture, and the HIV Interface: evidence from lakeshore border communities in Buliisa District, Uganda. J Tropical Medicine.

[67] Singer M (1994). AIDS and the health crisis of the U.S. urban poor; the perspective of critical medical anthropology. Soc Sci Med (1982).

