# Supplementary material for: Alcohol use, intimate partner violence, and HIV sexual risk behavior among young people in fishing communities of Lake Victoria, Uganda
Source: BMC Public Health. 2021 Mar 19;21:544. doi: 10.1186/s12889-021-10595-1 (PMC7978165; doi:10.1186/s12889-021-10595-1)
Supplement: Supplementary file 1 — Additional file 1. [file 12889_2021_10595_MOESM1_ESM.docx]

**Appendix 1: Survey Questionnaire (v6)**

# Section A: Preliminary Information

| **NO** | **QUESTIONS** | **CODING CATEGORIES** | **SKIPS/FILTERS**  **COMMENTS** |
| --- | --- | --- | --- |
| a1 | Identification Number (must be unique) | District code /sub-county code/ interviewer id / serial number | Tablet program |
| a2 | Date of Interview (auto) | dd/mm/yyyy |  |
| a3 | Interviewer ID & Name | Select ID & Name from list | Tablet program |
| a4 | Interviewer: Record Language of Interview | 1 = Luganda  2 = English  3 = Lusoga  4 = Lusamia  5. Others |  |
| a5 | District | Mukono = 1  Namayingo = 2 |  |
| a6 | Sub-county | See Excel Sampling File |  |
| a7 | Parish | **See Excel Sampling File** |  |
| a8 | Village / Enumeration Area (EA) | **See Excel Sampling File** |  |

# Section B: Background Characteristics

| NO | **QUESTIONS** | **CODING CATEGORIES** | **SKIPS/FILTERS**  **COMMENTS** |
| --- | --- | --- | --- |
| b1 | Observe and record gender as  MALE OR FEMALE? | 1= Male  2 = Female | Observe and record |
| b2 | When were you born? (eligible if born after July 1996 and before June 2005) | Year of birth……… | Eligible years of birth are 1996-2005 |
| b3 | What is your age in complete years? | _ _ Years | 15-24 years are eligible |
| b4 | INTERVIEWER: Record Age category | 1=15-19  2=20-24 |  |
| b5 | Are you currently enrolled in school? | 1 = Yes  0 = No  9=Refused |  |
| b6 | What is the highest formal schooling you have completed? | 0= No formal education  1=Primary  2=Secondary  3=Higher  8=Don’t Know  9=Refused |  |
| b7 | In the last 12 months, have you done any work for which you received a payment, either in cash or in kind? | 1 = Yes  0 = No |  |
| b8 | What is your main source of livelihood? | 1 = Fishing  2 = Student  3 = Farming  4 = Petty Trade  5 = Others |  |
| b9 | What is your religion? | 1 = Catholic  2 = Anglican  3 = Pentecostal  4 = Muslim  5 = Others |  |
| b10 | What is your current marital status? | 1 = Never married  2 = Married  3 = Living Together  4 = Widowed  5 = Divorced  6 = Separated |  |
| b11 | Have you ever given birth to or fathered a child? | 0 = No  1 = Yes  9 = Refused | If No or Refused, GOTO b13 |
| b12 | How many living children do you have?  (Type Number) | ……… …… Children |  |
| b13 | What is your tribe or ethnicity? | 1=Samia  2=Muganda  3=Musoga  4=Others |  |

# Section C: Sexual Activity & Partners’ Characteristics

| NO | **QUESTIONS** | **CODING CATEGORIES** | **SKIPS/FILTERS**  **COMMENTS** |
| --- | --- | --- | --- |
| c1 | In the last 12 months, have you had a sexual partner? | 0 = No  1 = Yes  9=Refused | If No or Refused, GOTO C16 |
| c2 | What is the age of your current sexual partner? (make a closer guess) | _ _ Years  8 = Don’t Know Age  9 = Refused |  |
| c3 | What is the main occupation of your partner? | 0 = Not working  1 = Fishing  2 = Student  3 = Farming  4 = Petty Trade  5 = Others (don’t specify) |  |
| c4 | What is the highest level of education of your partner? | 0= No formal education  1=Primary  2=Secondary  3=Higher  8=Don’t Know  9=Refused |  |
| c5 | Is your partner more educated than you? | 0= No  1=Yes  8=Don’t Know  9=Refused |  |
| c6 | Do you live or stay together with your partner? | 0= No  1=Yes |  |
| c7 | How long have you been in a relationship with your current partner in complete years? | 1= Less than a year  2=1-3 years  3= 4 years or more |  |
| c8 | Are you formally married (culturally introduced, church wedding or wedding in a mosque)? | 0= Never married  1=Living together  2=Culturally Introduced  3=Wedded |  |
| c9 | Do you have children with your current partner? | 0=No  1=Yes  9=Refused | If No, or Refused, GOTO c11 |
| c10 | How many children? |  |  |
| c11 | Do you have children who do not belong to your current partner staying with you? | 0=No  1=Yes  9=Refused |  |
| c12 | Are you of the same tribe or ethnic origin as your current partner? | 0=No  1=Yes  8=Don’t Know  9=Refused |  |
| c13 | How often does your partner get drunk with alcohol? | 0=Never drinks alcohol  1= Never gets drunk  2= Sometimes  3=Often |  |
| c14 | Do you think your partner has other sexual partners apart from you? | 0=No  1=Yes  8=Don’t Know  9=Refused |  |
| c15 | Does your partner accuse you of having other sexual partners? | 0=No  1=Yes  8=Don’t Know  9=Refused |  |
| c16 | Do you have any other sexual partners? | 0=No  1=Yes  9=Refused |  |

# Section D: HIV Knowledge and Safer Sex Attitudes

Now I would like to ask you some questions about what you know about some things related to health.

| NO | **QUESTIONS** | **CODING CATEGORIES** | **SKIPS/FILTERS**  **COMMENTS** |
| --- | --- | --- | --- |
| d1 | Have you **ever** heard of HIV?  (HIV means a virus that causes AIDS) | 1 = Yes  0 = No |  |
| d2 | Have you **ever** discussed HIV with anyone close to you e.g. friend, relative parent / guardian? | 1 = Yes  0 = No  9=Refused |  |
| d3 | Can a person reduce their chance of getting HIV by not having sex? | 1 = Yes  0 = No  8= Don’t Know  9=Refused |  |
| d4 | Can a person reduce their chance of getting HIV by using condoms when having sex? | 1 = Yes  0 = No  8= Don’t Know  9=Refused |  |
| d5 | Can a healthy-looking person have HIV or AIDS? | 1 = Yes  0 = No  8= Don’t Know  9=Refused |  |
| d6 | Can a mother with HIV or AIDS pass HIV to her unborn baby? | 1 = Yes  0 = No  8= Don’t Know  9=Refused |  |
| d7 | Are there medicines that people with HIV or AIDS can take to help them live longer? | 1 = Yes  0 = No  8= Don’t Know  9=Refused |  |
| d8 | Can male circumcision help prevent HIV infection?  Circumcision is the removal of the foreskin from a penis. | 1 = Yes  0 = No  8= Don’t Know  9=Refused |  |
| d9 | A woman is justified to refuse sexual intercourse with her partner if she knows that he has sex with other women | 1 = Yes  0 = No  8= Don’t Know  9=Refused |  |
| d10 | A woman is justified to ask her partner to use a condom if she knows that he has an STI | 1 = Yes  0 = No  8= Don’t Know  9=Refused |  |

# Section E: Alcohol use (AUDIT Tool)

“Now I am going to ask you some questions about your use of alcoholic beverages during this past year.” Explain what is meant by “alcoholic beverages” by using local examples of beer, alcohol sachets, etc (show the card with alcohol images). Questions e1-8 focus on the past 12 months and questions e9-10 are beyond the last 12 months (lifetime).

| **NO** | **QUESTIONS** | **CODING CATEGORIES** | **SKIPS/FILTERS**  **COMMENTS** |
| --- | --- | --- | --- |
| e1 | How often do you have a drink containing alcohol? | 0 = Never  1 = Monthly or less  2 = 2-4 times a month  3 = 2-3 times a week  4 = 4 or more times a week | If 0 (Never), GOTO e9-e10 |
| e2 | How many standard drinks containing alcohol do you take on a typical day when drinking?  (Use cards with pictures of drinks and show to the respondent. Ask if it is about sachets, bottles.) | 0 = 1 or 2 drinks  1 = 3 or 4 drinks  2 = 5 or 6 drinks  3 = 7, 8 or 9  4 = 10 or more |  |
| e3 | How often do you have six or more drinks on one occasion? | 0 = Never  1 = Monthly or less  2 = Monthly  3 = Weekly  4 = Daily / almost daily | If Total Score for Questions e2 and e3 = 0, GOTO Questions e9 - e10 |
| e4 | During the past year, how often have you found that you were not able to stop drinking once you had started? | 0 = Never  1 = Monthly or less  2 = Monthly  3 = Weekly  4 = Daily / almost daily |  |
| e5 | During the last year, how often have you failed to do what was normally expected of you, because of drinking? | 0 = Never  1 = Monthly or less  2 = Monthly  3 = Weekly  4 = Daily / almost daily |  |
| e6 | During the past year, how often have you needed a drink in the morning to get yourself going after a heavy drinking session? | 0 = Never  1 = Monthly or less  2 = Monthly  3 = Weekly  4 = Daily / almost daily |  |
| e7 | During the last year, how often have you had a feeling of guilt or remorse after drinking? | 0 = Never  1 = Monthly or less  2 = Monthly  3 = Weekly  4 = Daily / almost daily |  |
| e8 | During the past year, have you been unable to remember what happened the night before because you had been drinking? | 0 = Never  1 = Monthly or less  2 = Monthly  3 = Weekly  4 = Daily / almost daily |  |
| e9 | Have you or someone else been injured as a result of your drinking? | 0 = No  2 = Yes, but not in the past year  4 = Yes, during the past year |  |
| e10 | Has a relative or friend or a doctor or another health worker been concerned about your drinking or suggested you cut down? | 0 = No  2 = Yes, but not in the past year  4 = Yes, during the past year |  |
| **e11** | **Record total of specific items here =>** |  |  |
| **risk level intervention score**  zone 1 = alcohol education = **0-7**  zone 2 = simple advice (sa) = **8-15**  zone 3 = sa + brief counselling + monitoring = **16-19**  zone 4 = referral for diagnosis & treatment = **20-40** | | | |

# Section F: Intimate Partner Violence (IPV) Screening

Now I am going to ask you questions regarding your relationship with your current partner.

| **NO.** | **QUESTIONS** | **CODING CATEGORIES** | **SKIPS/FILTERS**  **COMMENTS** |
| --- | --- | --- | --- |
| f1 | In your lifetime or the last 12 months, have you been in a relationship with the opposite sex?  By relationship, I mean having someone of the opposite sex (boyfriend or girlfriend) you consider to be a potential marriage partner | 1 = Yes  0 = No  9 = Refused | 0, or 9, GO TO  Section G1 |
|  | **Hurt, Insult, Threaten & Scream (HITS) scale:** |  |  |
| f2 | How often did / does your partner physically **hurt** you? | 1 = Never  2 = Rarely  3 = Sometimes  4= Fairly often  5=Frequently |  |
| f3 | How often did / does your partner **insult** you or talk down to you? | 1 = Never  2 = Rarely  3 = Sometimes  4= Fairly often  5=Frequently |  |
| f4 | How often did / does your partner **threaten** you with harm? | 1 = Never  2 = Rarely  3 = Sometimes  4= Fairly often  5=Frequently |  |
| f5 | How often did / does your partner **scream** **or curse** you? | 1 = Never  2 = Rarely  3 = Sometimes  4= Fairly often  5=Frequently |  |
| **Women Abuse Screening Tool (WAST-SF):** consists of first two questions. Full WAST scale covers 6 more questions. The questions should be asked to men as well. | | | |
| f6 | In general, how would you describe  your relationship with your partner? | 1= No tension  2= Some tension  3= A lot of tension |  |
| f7 | Do you and your partner work out  arguments with great difficulty, some difficulty or no difﬁculty? | 1= No difﬁculty  2= Some Difﬁculty  3= Alot of difficulty |  |
| f8 | Do arguments ever result in you feeling down or bad about yourself? | 1= Never  2= Sometimes  3= Often |  |
| f9 | Do arguments ever result in hitting, kicking, or pushing? | 1= Often  2= Sometimes  3= Often |  |
| f10 | Do you ever feel afraid / frightened by what your partner says or does? | 1= Never  2= Sometimes  3= Often |  |
| f11 | Has your partner ever abused you physically? | 1= Never  2= Sometimes  3= Often |  |
| f12 | Has your partner ever abused you emotionally? | 1= Never  2= Sometimes  3= Often |  |
| f13 | Has your partner ever abused you sexually? | 1= Never  2= Sometimes  3= Often |  |
| Abuse Assessment Screen (AAS): Yes and No responses | | | |
| f14 | In the **past 12 months**, were you emotionally or physically abused by your partner? | 1 = Yes  0 = No |  |
| f15 | In the last 12 months, has anyone or your partner forced you to have **sexual activities** against your will? | 1 = Yes  0 = No |  |
| Help-seeking for IPV | | | |
| f16 | For any of the experiences (sexual violence, physical violence or emotional violence), did you tell anyone or seek for help? | 1 = Yes  0 = No | If No, GOTO g1 |
| f17 | Whom did you tell or where did you seek for help?  *(multiple choices)* | 0 = Friends  1 = Parents  2 = Relatives  3 = Police  4 = Church  5 = CDO  6 = Probation Office  7 = Local Council  8 = Court  9 = VHT |  |
| f18 | Did you obtain help after reporting such occurrences? | 1 = Yes  0 = No |  |

# Section G: HIV-Risk Screening Instrument (HSI)

| **NO.** | **QUESTIONS** | **CODING CATEGORIES** | **SKIPS/FILTERS**  **COMMENTS** |
| --- | --- | --- | --- |
| g1 | In the past 12 months, have you had 2 or more sexual partners? | 1 = Yes  0 = No |  |
| g2 | Have you had sex (a man puts his penis into the vagina) with any of your sexual partners during the past 12 months? | 1 = Yes  0 = No |  |
| g3 | How often have you used a condom when having sex in the past 1 year? | 1 = Never / Sometimes  0 = Always / Not had sex |  |
| g4 | Have you ever had a sexually transmitted disease such as gonorrhea, syphilis, chlamydia, genital warts, or genital herpes? | 1 = Yes  0 = No |  |
| g5 | At any time in the past 1 year, have you ever given money or alcohol or drugs to anyone to have sex with you? | 1 = Yes  0 = No |  |
| g6 | Have you ever been given money or alcohol or drugs or fish by someone to have sex with them? | 1 = Yes  0 = No |  |
| g7 | Have you ever injected street drugs, steroids, or vitamins with a needle? Or used oral drugs such as Miraa, Bhang, Glue, Kuber, Mandrax, Cocaine, Heroin, or others? | 1 = Yes  0 = No |  |
| g8 | Have any of your sexual partners in the past 12 months ever injected street drugs, steroids, or vitamins with a needle? Or used drugs like Miraa, Bhang, Glue, Kuber, Mandrax, Cocaine, Heroin, or others? | 1 = Yes  0 = No / Dont know |  |
| g9 | Have any of your sexual partners in the past 1 year been men who have had sex with other men? | 1 = Yes  0 = No / Dont know |  |
| g10 | Has your sexual partner or partners ever had a sexually transmitted disease such as gonorrhea, syphilis, chlamydia, genital warts, or genital herpes? | 1 = Yes  0 = No / Dont know |  |

# Section H: Sexually Transmitted Infections

| **NO** | **QUESTIONS** | **CODING CATEGORIES** | **SKIPS/FILTERS** |
| --- | --- | --- | --- |
| h1 | During the last 12 months, have you had an **abnormal discharge** from your vagina or penis or experienced pelvic pain? (e.g. unusual smell, color, or texture). | 1=Yes  0=No  9=Refused |  |
| h2 | During the last 12 months, have you had an **ulcer or sore** on or near your vagina (woman) or groin area (man)? | 1=Yes  0=No  9=Refused |  |
| h3 | During the last 12 months, have you had **pain on urination**? | 1=Yes  0=No  9=Refused |  |
| h4 | In the last 12 months, did a doctor, clinical officer or nurse tell you that you had a sexually transmitted disease other than HIV? | 1=Yes  0=No  9=Refused |  |
| h5 | In the last 12 months, did you seek or receive treatment for sexually transmitted infections (STIs) excluding HIV care? | 1=Yes  0=No  9=Refused |  |

# Section I: HIV Prevention Practices

People try several things to reduce the risk of being infected with HIV and other STDs.

In the **PAST 3 MONTHS,** have you done any of the following to protect yourself from HIV and other STDs?

| **NO.** | **QUESTIONS** | **CODING CATEGORIES** | **SKIPS/FILTERS**  **COMMENTS** |
| --- | --- | --- | --- |
| i1 | Abstained from sex | 1 = Yes  0 = No |  |
| i2 | Had sex with only one partner | 1 = Yes  0 = No |  |
| i3 | Used condoms every time you had sex | 1 = Yes  0 = No |  |
| i4 | Discussed HIV with your sexual partner(s) | 1 = Yes  0 = No |  |

# Section J: HIV Testing

People are interested in knowing their HIV status. Some go for HIV testing while others do not. I would like to know your experience.

| NO. | **QUESTIONS** | **CODE CATEGORIES** | **SKIPS/FILTERS** |
| --- | --- | --- | --- |
| j1 | Do you know where to get an HIV test? | 1 = Yes  0 = No |  |
| j2 | Have you **ever** tested for HIV and received the results? | 1 = Yes  0 = No |  |
| j3 | In the last 12 months, did you test for HIV and got the results? | 1 = Yes  0 = No |  |
| j4 | In the last 3 months, did you test for HIV and get the results? | 1 = Yes  0 = No |  |
| j5 | Do you intend to have an HIV test in 3 months? | 1 = Yes  0 = No |  |
| j6 | GPS location (capture using tablet / device) |  |  |
|  | End of Interview: Thank you for participating in our survey | |  |
